# Seeing is believing? Comparing plant–herbivore networks constructed by field co‐occurrence and DNA barcoding methods for gaining insights into network structures

**DOI:** 10.1002/ece3.4860

**Published:** 2019-02-07

**Authors:** Chunchao Zhu, Dominique Gravel, Fangliang He

**Affiliations:** ^1^ State Key Laboratory of Biocontrol, School of Life Sciences Sun Yat‐sen University Guangzhou China; ^2^ ECNU‐Alberta Joint Lab for Biodiversity Study, Tiantong National Station for Forest Research, School of Ecology and Environmental Sciences East China Normal University Shanghai China; ^3^ Département de Biologie Université de Sherbrooke Sherbrooke Quebec Canada; ^4^ Department of Renewable Resources University of Alberta Edmonton Alberta Canada

**Keywords:** diet identification, DNA barcoding, molecular network, observation network

## Abstract

Plant–herbivore interaction networks provide information about community organization. Two methods are currently used to document pairwise interactions among plants and insect herbivores. One is the traditional method that collects plant–herbivore interaction data by field observation of insect occurrence on host plants. The other is the increasing application of newly developed molecular techniques based on DNA barcodes to the analysis of gut contents. The second method is more appealing because it documents realized interactions. To construct complete interaction networks, each technique of network construction is urgent to be assessed. We addressed this question by comparing the effectiveness and reliability of the two methods in constructing plant–Lepidoptera larval network in a 50 ha subtropical forest in China. Our results showed that the accuracy of diet identification by observation method increased with the number of observed insect occurrences on food plants. In contrast, the molecular method using three plant DNA markers were able to identify food residues for 35.6% larvae and correctly resolved 77.3% plant (diet) species. Network analysis showed molecular networks had threefold more unique host plant species but fewer links than the traditional networks had. The molecular method detected plants that were not sampled by the traditional method, for example, bamboos, bryophytes and lianas in the diets of insect herbivores. The two networks also possessed significantly different structural properties. Our study indicates the traditional observation of co‐occurrence is inadequate, while molecular method can provide higher species resolution of ecological interactions.

## 1 INTRODUCTION

Ecosystem functioning and stability are dependent upon who eat whom in the ecological network (Derocles et al., 2014; Montoya, Pimm, & Solé, 2006). Empirical food webs are not randomly organized but exhibit very specific structural properties influencing their dynamics (Jacquet et al., 2016). Ecological interaction networks are often characterized by their modularity, which describes the division of networks into groups (Olesen, Bascompte, Dupont, & Jordano, 2007) and nestedness, which depicts the tendency of specialists interacting with the subset of those species interacting with generalists (Bascompte, Jordano, Melián, & Olesen, 2003). These network characteristics representing resource partition and link organization have been proved to influence species coexistence and stability of community (Delmas et al., 2019; Thébault & Fontaine, 2010). However, rarely can the network of an ecosystem be fully reconstructed due to sampling incompletion and technical constraints on corroborating species interactions.

Various methods, including observation of diet interaction (Brousseau, Gravel, & Handa, 2018; Dyer et al., 2007; Novotny, Basset, Miller, Weiblen, et al., 2002) and identification of food resources in the guts of predators (Braley, Goldsworthy, Page, Steer, & Austin, 2010) have been used to construct food webs. Diet associations of insect herbivores have traditionally been reconstructed by observation from field survey and laboratory feeding or rearing trials (Dyer et al., 2007; Erwin, 1982; Forister et al., 2015; Novotny, Basset, Miller, Weiblen, et al., 2002). Observational methods are accessible and relatively fast to perform, which is a strong asset particularly in species‐rich communities, but they are contingent on sampling conditions, leading to incomplete or even unreliable data. For instance, fogging of target trees has been widely used to collect data on plant–insect interactions and explore host specificity of insect in forest community (Burns, Taylor, Watson, & Cunningham, 2015; Erwin, 1982; Frederick & Gering, 2006). While easy to apply, this method is potentially unreliable due to high proportion of nonfeeding tourists (Stork, 1987). Subsequently, feeding or rearing trails are more preferred to construct and validate feeding associations of plants and insect herbivores (Dyer et al., 2007; Novotny, Basset, Miller, Weiblen, et al., 2002). These are however time consuming and labor intensive for sorting a large number of foraging associations and they are also subject to bias because of the sensitivity of diet choice to external environments. Furthermore, the observational method tends to focus on insect herbivores from common woody plant species (Novotny, Basset, Miller, Drozd, & Cizek, 2002), thereby ignoring other rare plant species and thus rare interactions.

Another uncertainty associated with the observational method is taxonomic identification. To accurately classify a large number of insect herbivores to species level based on morphological traits is a huge challenge in food web study, particularly when cryptic insect species are involved (Derocles, Evans, Nichols, Evans, & Lunt, 2015). In many studies, insects are assigned to the lowest identified taxonomic level or morphospecies in constructing plant–insect herbivore networks (Dyer et al., 2007; Novotny, Basset, Miller, Weiblen, et al., 2002). However, the identification of many insect herbivores in these studies is not taxonomically fine enough to construct accurate networks. This problem of taxonomic resolution could affect the assessment of host specificity of insect herbivore and may lead to controversy (Dyer et al., 2007; Novotny, Basset, Miller, Weiblen, et al., 2002).

Molecular methods such as DNA barcoding and metabarcoding techniques are increasingly applied to food web studies with the development of sequencing technique (García‐Robledo, Erickson, Staines, Erwin, & Kress, 2013; Kartzinel, et al., 2015; Wirta et al., 2014) and contribute to solving the problem of low species resolution and diet identification efficiency. For instance, animal DNA barcoding COI (mitochondrial cytochrome *c* oxidase 1) has been widely used in identifying parasitoids for constructing feeding associations between hosts and parasitoids (Derocles et al., 2015, 2014; Šigut et al., 2017; Wirta et al., 2014). The use of COI marker in delimiting insect species has also been well established (Hajibabaei, Janzen, Burns, Hallwachs, & Hebert, 2006; Hebert, Cywinska, Ball, & DeWaard, 2003; Hebert, DeWaard, & Landry, 2010). It can not only help identify cryptic insect species and but also improve estimation of insect species diversity (Strutzenberger, Brehm, & Fiedler, 2011). COI marker can accurately identify cryptic species of parasitoids (Derocles et al., 2015) and detect feeding associations missed by the traditional rearing method (Wirta et al., 2014).

Plant DNA barcoding and metabarcoding have also been applied in reconstructing plant–herbivore networks by identifying plant residues within animal guts or feces (García‐Robledo et al., 2013; Jurado‐Rivera, Vogler, Reid, Petitpierre, & Gómez‐Zurita, 2009; Kartzinel et al., 2015). Short fragment chloroplast DNA such as *rbcLa* and *trnL* (UUA), which can be efficiently amplified for degraded plant genome DNA, are used in reconstructing plant–herbivore food webs (Jurado‐Rivera et al., 2009; Kartzinel et al., 2015; Navarro, Jurado‐Rivera, Gómez‐Zurita, Lyal, & Vogler, 2010). However, unlike animal barcoding COI, none of the chloroplast markers exhibits high genetic variation to discriminate plant species well (Jurado‐Rivera et al., 2009; Kartzinel et al., 2015; Navarro et al., 2010). Current studies find that ribosomal internal transcribed spacer 2 (ITS2) could efficiently detect and identify food plants in arthropods due to high evolutionary rates (García‐Robledo et al., 2013; Pumariño, Alomar, & Agustí, 2011). For instance, ITS2 correctly identifies more than 60% of rolled‐leaf beetle's food plants in the order Zingiberales to species level that is otherwise impossible (García‐Robledo et al., 2013). Although some studies have attempted to use multiple plant DNA markers to reconstruct plant–herbivore webs (García‐Robledo et al., 2013; Kajtoch, 2014; Nuwagaba, Zhang, & Hui, 2015), the application of DNA barcoding and metabarcoding methods for identifying food plants of herbivores is mainly based on single plant DNA marker (Erickson et al., 2017; Kartzinel et al., 2015; Navarro et al., 2010). This has greatly compromised the potential of these molecular methods in discriminating foraging associations at a high taxonomic resolution.

To explore the applicability and effectiveness of multiple DNA markers, we reconstructed and compared DNA‐based plant–Lepidoptera larval network with the network established using the tradition method in a 50 ha subtropical forest plot in south China. We analyzed species resolution of the molecular method and quantified bias in the identification of pairwise interactions between Lepidoptera larvae and their host plants using traditional observations of co‐occurrence and DNA analyses of larvae and their gut contents. We used animal DNA barcode COI gene to identify Lepidoptera larvae and three DNA markers for plants (*rbcLa*, *trnL *and ITS2). We were interested in addressing two questions: (a) How reliable are plant DNA markers and observation method in identifying plant diet species? Are plant taxa and links identified in the plant–Lepidoptera larval networks reconstructed by the observational and molecular methods significantly different? and (b) How would the differences of plant taxa and links between the two networks contribute to our understanding of network properties? We presumed that molecular analysis of interactions can identify more nodes due to its ability in identifying true feeding associations and also in discriminating cryptic taxa. As a consequence, we expected that the DNA‐based network would possess structural properties different from the traditional network due to the variation in species and links informed by the two techniques. Particularly, we expected that DNA‐based network had higher specialization than the observation‐based network due to a possible increase in the number of host plants that may be identified by the high resolution of the DNA markers.

## 2 MATERIALS AND METHODS

### 2.1 The samples of Lepidoptera larvae

This study was conducted in a subtropical forest located within Heishiding Nature Reserve, south China (111°53′E, 23°27′N; elevation range: 150–927 m). Annual precipitation in the reserve is about 1743.8 mm, 79% of which falls from April to September. Dry season occurs from October to March. This study was performed in a 50 ha plot where all tree stems with diameter at breast height >1 cm were mapped. There are in total 213 woody plant species (129 genera, 60 families) in the plot. The samples of Lepidoptera larvae were collected during the wet seasons of May–October, 2013 and April–October, 2014. We collected insect samples by fogging 1% pyrethrum toward the crown of focal trees with Swing Fogger N50 (Adis, Basset, Floren, Hammond, & Eduard, 1998), for 2.5 min per tree. Shade cloth was spread around the focal tree to capture the fallen insects for 2 hr after fogging. To reduce the possible effect of wind on sampling, we focused on trees with heights from 3 to 12 m. Insect samples were collected from 72 stems, one stem from each of 72 tree/shrub species belonging to 50 genera and 26 families (a subset of the total 213 tree/shrub species of the whole plot). These 72 species of plants comprised rare, common and abundant species in the plot and accounted for 80.33% of total tree abundance of the plot. Each month, 72 insect samples were fogged from one stem of each of the 72‐plant species. Trees that were fogged would not be resampled in the future.

Sampled insects from each stem were preserved in one plastic bottle filled with 100% ethanol to inactivate digestive enzymes in the field and preserve insect tissues (Post, Flook, & Millest, 1993). Samples were stored at −40°C freezer in the biological station prior to transporting to the laboratory by dry ice and stored again at −40°C or −80°C. Each Lepidoptera larva was then sorted from other insects in the sample and was photographed. Most individuals were <1 cm in length and were photographed using a Leica camera (model M205) under the microscope; larvae with larger than 1 cm were photographed using a Canon camera (model PC1438). In total, we sorted 2,860 individuals of Lepidoptera and Lepidoptera‐like larvae under the microscope.

### 2.2 Classification of Lepidoptera larvae

Mixed plant and Lepidoptera genomic DNA was extracted from preserved larval Lepidoptera samples using DNeasy Blood & Tissue Extraction kit (Qiagen, Hilden, Germany). If multiple morphologically identical late‐instar larvae were sampled from one tree, one representative individual was chosen to perform DNA extraction. For larva <1 cm length, entire body of larva was used to extract mixed genome DNA. For larva larger than 1 cm length, we used sterile scalpels and forceps to dissect the larva's gut and then used midgut tissue to extract the DNA. DNA extractions were used as a template for amplification of mitochondrial fragment cytochrome oxidase subunit I (COI). PCR amplification was performed by LA Taq DNA polymerase (Takara, Japan), with bovine serum albumin (20%) added to enhance PCR amplification yield. Primer sequences of COI genes and PCR annealing temperatures used are shown in Supporting Information Table S1. The PCR amplifications were conducted with a protocol consisting of 5 min at 4°C pre‐denaturing, 35 cycles with denaturing at 94°C (30 s), annealing at 54°C (30 s), extending at 72°C (1 min), and a final extension step at 72°C (10 min). All PCR products were visualized by 1% agarose gel electrophoresis and sequenced directly using the BigDye Terminator Sequencing kit by the Sanger sequencing method in ABI 3730.

All sequences were assembled and edited manually with Seqman software package (Lasergene 7.0 package, DNAstar Inc.). Poor‐quality sequences were discarded and low‐quality ends were manually trimmed. Filtered sequences are aligned in Clustal Omega ( https://www.ebi.ac.uk/Tools/msa/clustalo/). Previous studies showed that most Lepidoptera insects had lower intraspecific genetic divergence (0.17%–0.43%) and only a few Lepidoptera species had more than 2% genetic divergence across broad geographical regions that were attributed to cryptic species overlooked in current taxonomic system (Hebert et al., 2010). Besides, 2% or 3% threshold based sequence divergence is confirmed to distinguish insect species (and morphological species) (Šigut et al., 2017; Strutzenberger et al., 2011). As intraspecific genetic differentiation within a plot should be lower than across plots, Lepidoptera larvae sampled from local plot are delimited by 2% sequence divergence in Seqman software package (Lasergene 7.0 package, DNAstar Inc.). These OTUs are further confirmed in Mothur software V. 1.39.5 (http://www.mothur.org/) and blasted against the NCBI's nucleotide database (nt) using a BLASTn algorithm (Morgulis et al., 2008; Zhang, Schwartz, Wagner, & Miller, 2000).

Species and family taxonomic labels of each Lepidoptera larva were mainly assigned according to best‐hit taxa in BLAST result. Taxonomic information was referred to the NCBI taxonomy database (http://www.ncbi.nlm.nih.gov/taxonomy). Morphological families of 141 Lepidoptera species identified by photographed larvae were also used to confirm and correct the taxonomic labels identified by BLAST. These morphological families of Lepidoptera included Geometridae, Lymantriidae, Nolidae, Limacodidae, and Sphingidae. If taxonomic family of the best‐hit taxa and morphological family were consistent, we followed the taxonomic identification in the BLAST result. If they were different, we combined the morphological family and the taxonomic families of the top 5 best‐hit taxa to assign Lepidoptera species to the most likely taxonomic label. Only those Lepidoptera species reliably identified by molecular method were retained for further analyses.

### 2.3 Interaction networks of Lepidoptera larvae and host plants

We first established dietary associations using the observation of host plants where sampled Lepidoptera larvae were collected. We further established dietary associations using plant DNA barcoding of larval gut content to identify host plants. To choose suitable reliable primers, we investigated whether five plant DNA markers showed positive amplification and sequencing for all 213 woody plants in the plot. *RbcLa* of all 213 species and *trnL* of 205 species were successfully obtained while *trnH‐psbA* sequences of 189 species and *matK *sequences of 201 species were successfully obtained. ITS2 with high species resolution had low success rate of sequencing (113 of 213 woody plant species) due to multiple copies. Besides, comparing to the other two cpDNA markers, we found that* trnL*, *rbcLa, *and ITS2 had higher DNA amplification efficiency for the plant residues within larva guts. Thus, three DNA markers (*trnL*, *rbcLa,* and ITS2; see Supporting Information Table S1) were used to identify plant residues within larva guts.

PCR amplifications were conducted using the three DNA markers. Due to poor DNA quality and low primer binding efficiency, three pairs of PCR primers successfully amplified 50.1% larva sample. One dominant DNA band was obtained for each PCR product except for that 33 PCR productions appeared multiple DNA bands. All these PCR products were directly purified and sequenced in ABI 3730 sequencer. For all sequences, we checked chromatogram files, trimmed low‐quality ends of each sequence and retained those high‐quality sequences whose length was no <80% of the expected length. Seventeen *rbcLa* sequences, 10 *trnL* sequences and 53 ITS2 sequences were discarded from our analyses due to poor quality. We compared sequences using the BLASTn algorithm (Morgulis et al., 2008; Zhang et al., 2000) with a local plant DNA barcode database and the NCBI database, respectively. The local database includes all recorded 213 tree/shrub species from the 50 ha study plot. We identified the plants eaten by Lepidoptera larvae by referring to the taxa with the highest sequence similarity and DNA markers that can well distinguish target food plants from the others.

Based on matched Lepidoptera larvae identified by the two diet identification approaches, we reconstructed food webs based on the observational approach (the observation network) and food webs based on DNA barcoding (the molecular network).

### 2.4 The differences in nodes and links of molecular and observation networks

We compared the differences of nodes and links identified by two methods on species and genus levels. Further, we calculated the proportions of nodes and links exclusively and commonly identified by the two methods.

### 2.5 Reliability of plant DNA markers in diet identification

A total of 102 plant species or species complexes were identified from molecular data. We assessed the accuracy of diet identification by the combination of the three DNA markers and each marker at family, genus and species levels. If the sequence of one species can be distinguished from the other species in local plant database, the resolution of the species was assigned as 1 and otherwise was 0. A total of 15 species food plants in our data were reliably identified as bamboos, bryophytes, and lianas by BLAST. These nontree taxa lacked local reference sequences but can be distinguished from the local plant sequences. We conservatively assigned those taxa with 0.5 species resolution. Likewise, each food plant was assigned respective resolution value at the family and genus levels. Finally, we calculated the diet identification resolution of the three plant DNA markers and their combination at family, genus and species levels. The resolution of diet identification was averaged by individuals of food plants identified by molecular approach.

### 2.6 Accuracy of observation method in diet identification

For each Lepidoptera larva, by comparing sampled host obtained by field observation to corresponding food plant(s) identified by molecular method, we assessed the accuracy of diet identification of the observation method at family, genus and species levels. As well, diet identification of the observation method was assessed by each of three plant DNA markers.

### 2.7 The diet mismatching of molecular and observation methods

We explored what would cause a larva collected from a tree was not confirmed by molecular method to feed on the tree. The bias of observation and molecular methods should contribute to this diet mismatching. False positive identification of interactions by observation of co‐occurrence could arise from those larvae occasionally dropped from nearby neighboring trees and those that happened to disperse from distant neighboring trees. Because 2 × 2 m size of the shading cloth was used to collect dropped larvae at fogging, we used 2 m distance to distinguish near (≤2 m) and far (>2 m) neighbors and calculated their contribution to the sample bias. The mismatch between the field‐sampled hosts and the “additional nontree taxa” including bamboos, bryophytes, and lianas that were reliably identified to genus at least by our three DNA markers were considered as the bias of the observational method. The bias of observational method was thus attributed to the larvae on the sample tree that fed on near/far neighbors or the unsampled nontree taxa. In contrast, bias in the DNA method occurs if plant residues could not be identified to species. If a food plant of a Lepidoptera larva was ambiguously identified by molecular method to species level but found within the neighbors 2 m of the fogged tree, this diet mismatching was attributed to the bias of observation method. In addition, although low species resolution taxa identified by molecular method can be found in the far neighbors 2 m away from the focal tree, this diet mismatching of the two methods was attributed to bias of the molecular method.

### 2.8 The effect of sampling efforts on diet identification

We tested how the foraging probability of Lepidoptera species detected by molecular method varied with the number of observed occurrences of Lepidoptera species on host plant species with logistic regression. The detection of an interaction from the gut residues by the molecular method was recorded as 0 (no, the foraging was not confirmed) or 1 (yes, confirmed) and modeled as a function of the number of observed occurrences of Lepidoptera species on the corresponding host plant species.

### 2.9 Network structural properties of molecular network and observation network

To investigate how diet detectability and bias of network construction method impacted network properties, we compared the molecular network and the observation network at the whole network level. To detect how resolution of molecular method may contribute to the difference between the two networks, we further compared individual‐level molecular network constructed by one DNA marker (*RbcLa*, *trnL*, or ITS2) and the combinations of two DNA markers (*RbcLa + trnL*, *RbcLa + ITS2*, or *trnL* *+ ITS2*) with the individual‐level observation network, respectively. The compared individual‐level molecular network and individual‐level observation network were constructed by the same insect sample.

The following qualitative network metrics were calculated using the *bipartite* package (Dormann, Fründ, Blüthgen, & Gruber, 2009): network specificity (Blüthgen, Menzel, & Blüthgen, 2006), interaction evenness, generality, vulnerability, and nestedness (Almeida‐Neto, Guimarães, Guimarães, Loyola, & Ulrich, 2008). Quantitative network metrics were also calculated; they included quantitative generality, quantitative vulnerability, quantitative nestedness (Almeida‐Neto & Ulrich, 2011), and quantitative modularity (Beckett, 2016). Network specificity (Blüthgen et al., 2006) and interaction evenness (Shannon's evenness of interactions) describe niche partition pattern at the network level. Quantitative generality/vulnerability, calculated as the mean effective number of interactive partners per insect/plant weighted by their marginal totals, represent niche partition at each trophic level. Nestedness and modularity capture network‐level link organization (Delmas et al., 2019). Nestedness calculated based on NODF method (Almeida‐Neto et al., 2008; Almeida‐Neto & Ulrich, 2011), occurs when the diets of a specialist species are a proper subset of a generalist species (Bascompte et al., 2003). Modularity measures the extent to which a network is divided into small subwebs (Delmas et al., 2019; Olesen et al., 2007). Quantitative modularity was calculated using LPAwb+ algorithm (Beckett, 2016).

To control the effect of network size on structural properties, each observed network metric for the observational network was compared to that of 1,000 random subwebs of the molecular network with equal number of nodes. Based on the 1,000 randomized molecular networks, we calculated mean value and 95% confidence interval for each network metric.

## 3 RESULTS

### 3.1 Species identification of Lepidoptera larvae

In total, we delimited 446 OTUs based on 2,279 high‐quality COI representative sequences. Having filtered out 40 problematic OTUs, which included eight OTUs identified as different Lepidoptera families and 32 OTUs identified as the other insect orders, we successfully identified 2,235 Lepidoptera individuals to 406 OTU species. By BLAST, the 406 OTU species of Lepidoptera delimited by sequence divergence matched 350 Lepidoptera species in NCBI database. The remaining 56 species were identified to the families of Lepidoptera. These 406 species of Lepidoptera were assigned to 36 families of Lepidoptera, and they were the Lepidoptera larvae analyzed in this study.

Plant residues in the guts of 795 out of 2,235 Lepidoptera individuals (35.6%) were identified by plant DNA markers. Plant sequences of 671 from the 795 Lepidoptera individuals were then successfully obtained by the primers of at least one DNA fragment. The sequences of the 671 Lepidoptera larvae included 507 *rbcLa *sequences and 373 *trnL* sequences and 198 ITS2 sequences (Table 1). Thus, food residues in the guts of the 795 Lepidoptera larvae were identified using DNA markers.

**Table 1:** The resolution of diet identification to family, genus and species levels by three plant DNA markers

Plant DNA barcodes	Averaged by diet individuals			Number of sequences	Amplicon length (bp)
	Family (%)	Genus (%)	Species (%)		
*rbcLa*	100	88.4	70.5	507	~540
*trnL*	100	86.1	68.9	373	~500
Internal transcribed spacer 2	100	100	93.7	198	350–400
Three markers	100	97.6	77.3	671	

### 3.2 The differences in nodes and links of molecular and observation networks

We found 239 Lepidoptera species associated with 72 host plant species based on the field observations but associated with 102 plant species (species complexes) based on the molecular method. More links were however detected by the observational method (546 plant species vs. 518 plant genera) than by the molecular method (408 vs. 396). Also, more unique links were detected in the observation network than the molecular network (Table 2). The molecular and observational networks only shared 98 links on the plant species level and 112 links on the genus level (Table 2). After excluding the unique plant nodes from each network, the two networks shared a higher percentage of links (Table 2).

**Table 2:** Number of nodes and links for the matched 795 caterpillars identified by the traditional observation method and the DNA barcoding method

	Group	T+M+ (%)	T+M− (%)	T−M+ (%)	Total
Nodes	Host genera	47 (47.4)	3 (5.3)	35 (47.4)	85
	Host species	56 (47.5)	16 (13.6)	46 (40)	118
Links (shared nodes)	Host genera	112 (16.8)	393 (59.1)	160 (24.1)	665
	Host species	98 (16.5)	358 (60.3)	138 (23.2)	594
Links (all nodes)	Host genera	112 (14.0)	406 (50.6)	284 (35.4)	802
	Host species	98 (11.4)	448 (52.3)	310 (36.2)	856

Notes

All statistics were calculated from interactions between Lepidoptera species and host plant species and genera, respectively. “T+M+” denotes nodes/links identified commonly by the observation and molecular methods. “T+M−” denotes nodes/links identified exclusively by the observation method. “T−M+” denotes nodes/links identified exclusively by the molecular method.

**Figure 1:**
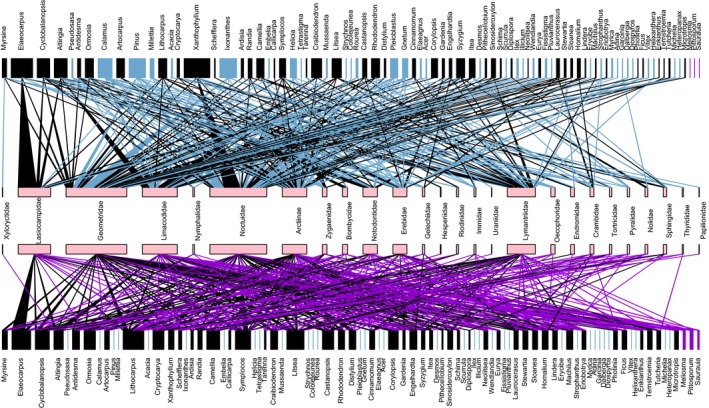
Food webs constructed with the observational method (the lower panel) and molecular method (the upper panel). All host plant genera found by the two methods are listed on the graph. To avoid cluttering, family (rather than genus) names of Lepidoptera larvae are listed. Unique plant genera (links) found in observation and molecular networks are marked in dark violet and sky blue, respectively. The nodes and links of common genera found by both methods are shown in dark. Box size in the networks is proportional to node abundance. Line width of links represents interaction strength

The majority of 72 sampled trees were correctly identified by the molecular method, but 16 of them (belonging to 11 genera) were not recovered by the DNA markers (see Figure 1 and Supporting Information Table S2). Of these 11 plant genera, three genera (*Meliosma, Pittosporum*, and* Saurauia*) that comprised three species were exclusively detected by the observation network but the other eight genera (comprising 13 species) were detected by both methods. The 13 tree species undetected by the molecular method may be due to a low congeneric variation in DNA markers or local morphological misidentification. For instance, of four species belonging to plant genus *Cyclobalanopsis *(species‐rich family Fagaceae), two species (*C. bambusaefolia* and *C. fleuryi*) were assigned as one species complex (*C. bambusaefolia*) and the other two species (*C. chungii* and *C. hui*) were assigned as another species complex (*C. chungii*) by molecular method due to low intraspecific variation (Table S3). Three sampled tree species (*Symplocos anomala, S. congesta*, *S. laurina*) were not detected by molecular method, while other three species of the same genus (*S. adenophylla*, *S. lancifolia*, *S. wikstroemiifolia*) were successfully detected (Table S3).

The molecular method detected more food plants (46 unique species/species complexes and 35 unique genera) than the observational method (16 unique species and three unique genera, see Table 2). There were 46 species (species complexes) exclusively detected by molecular methods, of which 15 species (“additional nontree taxa”) belonged to bamboos, lianas (*Calamus*, *Millettia*, *Tetrastigma*, *Rourea*, *Gnetum*, *Strychnos, Acacia* and *Erycibe* genera) and epiphytic or parasitic plants (*Cololejeunea*, *Pluvianthus*, *Scurrula* and *Helixanthera *genera; Figure 1 and Supporting Information Table S3).

Besides, we found that larval species of Lasiocampidae, Geometridae, Limacodidae, Noctuidae, Arctiinae, Lymantriidae were relatively abundant (Figure 1). In the molecular network, Noctuidae species and *Pinus *species, Geometridae species and *Ixonanthes *species, Noctuidae species and *Xanthophyllum* species interacted strongly with each other (Figure 1). No such (or such strong) interactions were detected in these Lepidoptera larvae in the observation network (Figure 1).

### 3.3 The reliability of plant DNA markers in diet identification

Among the three plant markers, the resolution of ITS2 marker was highest at the family, genus and species levels (100%, 100% and 93.7%; Table 1), followed by *rbcLa* (100%, 88.4% and 70.5%; Table 1) and *trnL* marker (100%, 86.1% and 68.9%; Table 1).

*RbcLa *had higher resolution in most plant families, but *trnL* had higher resolution in plant family Araliaceae and genus *Litsea *and *Neolitsea*. Because of multiple copies of ITS2 locus, we did not successfully obtain DNA sequences of plant genera *Machilus*, *Camellia*, *Craibiodendron*, *Desmos, *and *Cryptocarya *without supplement cloning of PCR production. Excluding the failed sequences of one or two loci, the resolution of diet plants identified by combinations of three DNA markers was very high at family, genus and species levels (100%, 97.6%, and 77.3%; Table 1). Further, *rbcLa* had higher diet recovery rates than *trnL* and ITS2 markers (Table 1).

### 3.4 The accuracy of the observation method in diet identification

Out of 795 Lepidoptera larvae whose diets were reliably identified by the molecular method, we found sampled trees of 228, 226 and 204 Lepidoptera larvae were consistent with the plants identified by molecular method at family, genus, and species levels. A total of 132 Lepidoptera larvae were confirmed to feed on sampled trees using *rbcLa* barcode. A total of 106 Lepidoptera larvae were confirmed to feed on sampled trees using *trnL* barcode. A total of 53 Lepidoptera larvae were confirmed to feed on sampled trees using ITS2 barcode.

### 3.5 Diet mismatching of molecular and observation methods

On the species level, only 25.7% Lepidoptera larvae (Table 3) were confirmed to feed on the tree where the larvae were collected. Diet mismatching of 62.2% Lepidoptera larvae identified by two methods was attributed to sampling bias of observation method (Table 3). Of this bias, 10.7% Lepidoptera larvae (85 individuals) were found to come from those feeding on near neighboring trees within 2 m around the sampled trees, 40.1% Lepidoptera larvae (319 individuals) mainly feeding on far neighboring trees 2–5 m away from the sampled trees and 11.4% Lepidoptera larvae (91 individuals) foraging bamboos, bryophytes, and lianas (Table 3). The technical bias of DNA markers led to 12.1% Lepidoptera larvae (96 individuals) having different food plants and sampled plants (Table 3). Overall, the bias (83.8%; Table 3) of the observational method contributed more to diet mismatching at species level than that of the molecular method (16.2%; Table 3).

**Table 3:** Difference between food plants identified by the traditional observation method and the molecular method at the species level

	Larvae feeding on sampled plants	Sampling bias of traditional method			Resolution bias of molecular method
Source of diet mismatch		Near neighbors	Far neighbors	Nontree taxa	Low‐resolution taxa
Number of larvae	204	85	319	91	96
Percentage of larvae	25.7%	10.7%	40.1%	11.4%	12.1%
Contribution of each method to diet mismatch		83.8%			16.2%

Notes

“Near neighbors” are those trees <2 m away from the sampled trees. “Far neighbors” are those trees >2 m away from the sampled trees. “Nontree taxa” refer to plant taxa identified by molecular method that are bamboos, lianas, bryophytes, and parasitic plants. “Low‐resolution taxa” denotes the food plants that were ambiguously identified to species by DNA makers.

### 3.6 The effect of sampling efforts on diet identification

**Figure 2:**
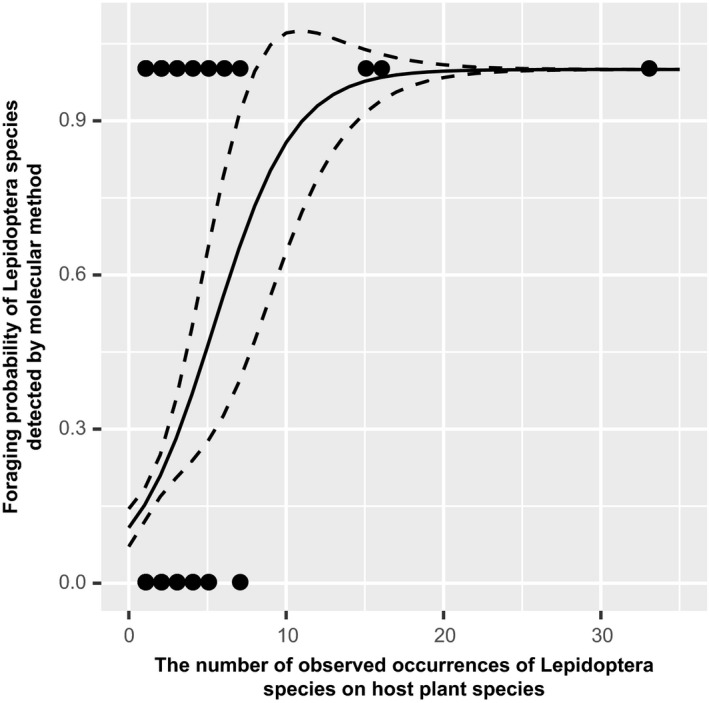
The relationship between the foraging probability of Lepidoptera species detected by molecular method and the number of observed occurrences of Lepidoptera species on host plant species. The fitted logistic regression model is as follows: $y=\frac{\exp\left(-2.11+0.39x\right)}{1+\exp\left(-2.11+0.39x\right)}$. The regression coefficient 0.39 is highly significantly different from 0 (*p* = 1.75 × 10^−4^).

The probability of Lepidoptera larvae feeding on sampled trees significantly increases with the number of observed occurrences of Lepidoptera species on host plant species (Figure 2). Predicted by the fitted logistic regression models, when the observed occurrences of Lepidoptera species on host plant species are more than 20 times, Lepidoptera larvae indeed have high probability (*p* = 0.99) feeding on sampled trees as confirmed by the molecular method (Figure 2). This suggests that to obtain reliable plant–insect herbivore associations based on observation method, a plant–insect herbivore interaction requires, on average, 20 observations in the field.

### 3.7 Network structure properties of the molecular and observational networks

**Figure 3:**
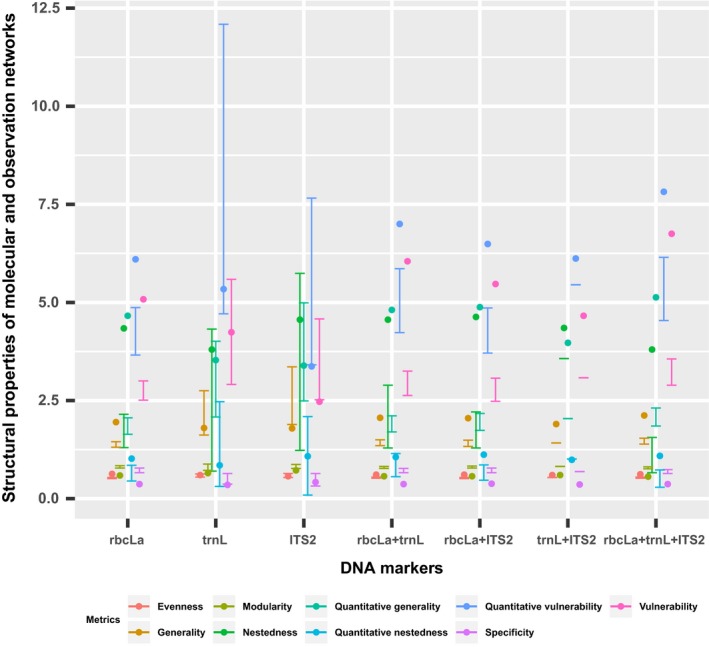
The comparison of network structural properties between the molecular network constructed by different DNA markers and the observation network based on fogging method. The error bar indicates the confidence interval of each network metric which was calculated based on the 1,000 randomized networks sampled from the molecular network constructed by different DNA markers. The point indicates the network metrics calculated based on the observation network. Due to that the molecular network constructed by two DNA markers internal transcribed spacer 2 + *trnL* has the same network size as the corresponding observation network, the value of each network metric instead of the confidence interval is shown

The observation network had significantly different qualitative and quantitative properties, even after controlling for network size, from the molecular network (Table 4). At the network level, the molecular network based on three DNA markers had higher specificity, lower interaction evenness, lower nestedness, and higher modularity than the observation network (Table 4). On each trophic level, the molecular network based on three DNA markers exhibited significantly lower vulnerability and generality (Table 4). As well, consistent network structural differences were found when comparing individual‐level molecular networks constructed by the combinations of two DNA markers or one DNA marker *rbcLa* and individual‐level observation networks constructed by fogging method (Figure 3). However, no significant difference was detected in some network properties such as quantitative generality and nestedness when comparing individual‐level molecular networks constructed by one DNA marker *trnL* or ITS2 and the individual‐level observation networks constructed by fogging method (Figure 3). Network properties of individual‐level molecular networks constructed by combinations of two DNA markers were more close to the molecular network constructed by the three DNA markers (Figure 3).

**Table 4:** Structural properties of molecular network and observation network at the species level

Network properties	Observation network	Mean of randomized molecular networks (95% confidence interval)
Qualitative network specificity	0.37^a^	0.70 (0.64, 0.74)
Qualitative interaction evenness	0.62^a^	0.54 (0.52, 0.55)
Qualitative generality	2.12^a^	1.46 (1.39, 1.54)
Qualitative vulnerability	6.75^a^	3.23 (2.89, 3.56)
Quantitative generality	5.13^a^	2.08 (1.85, 2.31)
Quantitative vulnerability	7.82^a^	5.45 (4.54, 6.15)
Qualitative nestedness	3.80^a^	1.14 (0.66, 1.56)
Quantitative nestedness	1.09^a^	0.55 (0.29, 0.73)
Quantitative modularity	0.56^a^	0.78 (0.76, 0.81)

a The observation network is significantly different from the metric averaged from 1,000 randomized networks of the molecular network at *p < *0.05.

## 4 DISCUSSION

Understanding plant and insect herbivore interaction have important implications for pest management and biodiversity conservation, for instance, food web analyses allow us to identify species critical to the stability of the network. These applications of plant–herbivore networks critically depend on reconstruction of accurate and unbiased interaction networks. However, how one may reconstruct complete and high resolved plant–insect herbivore networks is a question that has not been satisfactorily answered, particularly in species‐rich tropical and subtropical forests due to labor‐intensive feeding trails and the taxonomic challenge in identifying diverse morphological species. Our study showed that molecular techniques offer promises in reconstructing more accurate plant–herbivore networks than the traditional field‐based observation and laboratory‐rearing approach. By using three plant DNA barcodes and local plant reference database, we showed that the molecular approach achieved a higher efficiency in identifying diets than the traditional approach and unambiguously resolved almost 80% diet interactions on the species level (Table 1). In contrast, the traditional approach was more biased and the accuracy of its diet identification varied with the number of observed occurrences of insects on plants (Figure 2). The higher accuracy, efficiency and less labor intensive of molecular approach in identifying both insect herbivores and host plants, as shown in this study, suggest the great potentials of the method, alternative to the traditional method, in reconstructing reliable and complete plant–insect herbivore interaction networks.

### 4.1 Food identification by the molecular and observation methods

With the development of DNA barcoding techniques, molecular approaches have started to attract attention of ecologists for studying ecosystem networks (Derocles et al., 2015, 2014; García‐Robledo et al., 2013; Wirta et al., 2014). For example, DNA markers have been used to reconstruct plant–herbivore interaction networks (Braley et al., 2010; García‐Robledo et al., 2013; Jurado‐Rivera et al., 2009; Navarro et al., 2010), plant–leaf miner–parasitoids networks (Derocles et al., 2015) and plant–pollinator networks (Wilson, Sidhu, Levan, & Holway, 2010). In this study, DNA barcoding technique was used to reconstruct plant–insect herbivore feeding networks. Our results showed three DNA makers resolved 77.3% diet plants of insect herbivores to species (Table 1) which improved 61.6% identification rate achieved in a previous study (García‐Robledo et al., 2013). As well, our results showed that at the species level, the three DNA barcoding markers (*rbcLa, trnL,* and ITS2) allowed to recover 35.6% diet interactions. This result is rather remarkable when compared to other studies that used traditional rearing experiments and was only able to recover a much smaller fraction (<5%) of diet interactions (Dyer et al., 2007; Novotny, Basset, Miller, Drozd, et al., 2002). Besides, our study showed that the molecular approach was able to detect those food plants including bamboos, bryophytes, and lianas that were otherwise overlooked by the traditional observational method (Figure 1 and Supporting Information Table S2). These suggest plant DNA barcoding is feasible and efficient in capturing plant–herbivore associations.

*RbcLa* and *trnL* markers exhibited 100% and 96% positive amplification for the 213 local woody plant species studied here and had higher diet recovery rates than other markers. *RbcLa* had higher species resolution than *trnL* but lower species resolution than ITS2 (Table 1). The combination of *rbcLa *and *trnL *recovered diet associations for more Lepidoptera larvae and identified more plant species than the other two combinations of the three DNA markers (Table S4). We recommend using *rbcLa* marker to approximate plant–insect herbivore interactions and the combination of at least two DNA markers to construct high‐resolution diets. The combinations of two markers *rbcLa + trnL* and *rbcLa + ITS2* performed better than the *trnL* *+ ITS2*.

Despite the high effectiveness of the molecular method for identifying host plants, it is worth noting that many unique links in our data were not recovered by molecular approach (Table 2). As shown in Table 3, this problem is mainly due to the sampling bias of the observation method. The traditional method usually requires feeding trails to exclude tourist insects (Dyer et al., 2007; Novotny, Basset, Miller, Drozd, et al., 2002; Novotny, Basset, Miller, Weiblen, et al., 2002). Feeding was not conducted in this study which may lead to “false positive” links (thus an excessive number of links in the observation network, i.e., the links under the T + M− column in Table 2). As well, we found that the probability of observing plant–herbivore interactions depends on the frequency of their co‐occurrence (Figure 2).

When further examining the causes of the bias of the observation method (by looking at the spatial location of fogged trees in the plot; Supporting Information Figure S1), we found that the bias mainly aroused from dispersal behavior of Lepidoptera larvae from far neighboring trees plus occasional drops of Lepidoptera larvae from near neighboring trees (Table 3). Depending on the host species and larvae density, newly hatched larvae can spin off host plants by ballooning (Berger, 1992). Possibly triggered by the shortage of plant resources and the risk of predation, third instar and late‐instar larvae will also leave their natal plants to new plants or pupation sites (Berger, 1992). Bigger larvae with crawling ability are supposed to have better mobility than the smaller ones and thus may contribute more to false‐positive identification in the observation network. However, this seemed not to happen in our study because body size was not found to significantly affect diet identification of Lepidoptera larvae (logistic regression coefficient of body size = −0.04, *p* = 0.70).

To summarize, the observational method generates false positives because of spurious co‐occurrence. The molecular method is limited to observed co‐occurrences; therefore, interactions with rare species are difficult to document. Feeding trials can overcome this limitation, but they are sensitive to the context of the experiment and may draw interactions among species that are not co‐occurring.

### 4.2 Structural properties of molecular and traditional observation networks

At network level, all qualitative and quantitative metrics in the observation network in our study are significantly different from that of the standardized molecular networks (Table 4). This result indicates the observation network is not a random subset of the molecular network.

False‐positive interactions may lead to the observation network to have significantly biased network properties due to that diet associations were only established based on field observation without confirmatory feeding trails. After removing low‐frequency links (≤2) from an observation network that was constructed using all Lepidoptera larvae, we found that nestedness and modularity of the network were approximately similar to that of the molecular network (Supporting Information Figure S2). This indicates that bias of the observation method may be mainly caused by rare interactions and can be reduced by increasing sampling intensity.

Some factors related to the construction of molecular network can contribute to the network structural difference between the two networks. First, we found that the molecular method can detect food plants that were not sampled by the observation method, for example, those bamboos, bryophytes, and lianas (Supporting Information Tables S2 and S3). Second, the use of the partial sample (671 representative larvae) to reconstruct diet associations of 795 larvae by molecular method could also contribute to the difference in network structure between the two types of networks. Third, the technique bias associated with molecular method could also be a factor (Table 3). We found the difference in network structure between the molecular and observation networks increased with the application of multiple DNA markers (Figure 3). Thus, though partial plant nodes were not completely resolved in our molecular network, the pattern of such network structural differences between the two networks remains reliable.

In addition, the decreased sample size and increased delectability of new plant species may also increase specificity in the molecular network (Table 4). Increased resolution has been found to result in higher interaction specialization in DNA‐based host–parasitoid networks than the morphologically identified network by rearing method (Kaartinen, Stone, Hearn, Lohse, & Roslin, 2010; Smith et al., 2011). Comparing to those networks constructed by fogging method, higher specificity is also detected in our molecular networks constructed by two or three DNA markers (Figure 3). Our results of higher modularity and lower nestedness detected by the molecular network (Table 4) are consistent with the previous studies showing higher modularity and lower nestedness in the DNA‐based network than in the morphological‐based network (Derocles et al., 2015, 2014). Moreover, increased interaction evenness in our observation network (Table 4) may arise from more links resulting from false positive foraging associations. Taken together, the observational method tends to bias network characteristics while molecular approach can improve the qualitative and quantitative structural properties of networks. DNA barcoding is a useful method to reconstruct ecological networks and can enhance our understanding of food webs structure and dynamics.

### 4.3 Limitations and future improvements of plant DNA barcodes in food identification

As evident from this study, the recovery rate of food resources of Lepidoptera larvae by the molecular method is still low (35.6%). Low amplification efficiency (50.1%) of digested plant residues is mainly the constraint factor of molecular diet identification. Thus, to improve the recovery rate using DNA markers with small sample size, one urgent issue is to increase DNA amplification for digested plant residues. Low amplification efficiency may be caused by poor quality and low concentration of incomplete genome DNA extracted from digested plant residues. Small body size and longtime of digestion can reduce success of diet identification due to retaining less plant residues (Pumariño et al., 2011). Further, about 10% PCR products detected by gel electrophoresis were canceled sequencing due to low concentration. Thus, low concentration of PCR products also contributed to failure of diet identification. Though almost all PCR productions appeared one dominant DNA band, both multiple same length plant cpDNA fragments and multiple copies of nuclear ribosomal ITS2 may lead to failure of directly sequencing of plant PCR production. By analyzing sequence chromatogram files, we found that only seven of 17 poor‐quality *rbcLa *sequences and one of 10 poor‐quality *trnL* sequences had overlap peaks which may arise from mixed multiple sequences. A total of 53 sequences of ITS2 were also discarded due to poor quality. Thus, direct Sanger sequencing is not suited for complicated food mixtures and may lead to the low success of diet identification of Lepidoptera larvae.

To conclude this study, we offer a number of suggestions for improving the use of molecular method in reconstructing plant–herbivore networks. First, we recommend field sampling should focus on collecting late instars of larvae that have just fed on plants to improve diet identification rates. The longer after foraging, the poorer for the DNA markers to identify diet plants. Therefore, it is better to collect insects in the morning than in the afternoon because of longer foraging activity in the morning (Fitzgerald, Casey, & Joos, 1988). Second, because universal primers possibly have low binding efficiency with DNA of some food plants that have genetic variation in primer binding site, we may design a set of species‐specific primers for undetected sampled plants to increase sequence recovery rate. Using this approach, it has been shown that food plants can be well detected in the guts of Lepidoptera species using tomato‐specific ITS primer (Pumariño et al., 2011). Third, Sanger sequencing failure may arise from multiple copies or several fragments of food residues, thus we suggest using supplement metabarcoding sequencing (Evans, Kitson, Lunt, Straw, & Pocock, 2016; Kartzinel et al., 2015) for failed sequencing DNA fragments such as ITS2 to improve recovery rate and taxonomic resolution of food resources. One potential advantage is that DNA metabarcoding sequencing will likely improve identification efficiency for low concentration of plant residues. To fully reconstruct individual‐based food webs that have been partly recovered by Sanger sequencing in our study, individual metabarcoding sequencing for each Lepidoptera larva should be conducted.

The combination of DNA barcoding and sequencing techniques opens up a novel avenue for monitoring species diversity and trophic interactions essential for exploring mechanisms of species coexistence and community assembly. Further, due to that species richness and specificity in molecular network increases with the number of DNA markers, exploring networks with different species identification resolution should improve our understanding about complexity and community stability.

## AUTHORS CONTRIBUTION

CZ and FH designed the study. CZ conducted the experiments, data analysis and led the writing. CZ, DG, and FH contributed to the writing of the paper.

## Data Availability

Representative sequences of 406 Lepidoptera species were deposited in the GenBank nucleotide collection under accession numbers: MG986496–MG986632, MG986635–MG986720, and MK044350–MK044532. The accession number of representative sequences used to identify plant residues: MK120984–MK121080, MK121081–MK121143, MK121144–MK121180, MK125507, and MK125508. Plant–Lepidoptera larval interaction matrix constructed by molecular method, link similarity and dissimilarity identified by molecular and observation methods, and taxonomic information of 406 Lepidoptera species were deposited in Dryad Digital Repository (https://doi.org/10.5061/dryad.8q9v6mv).

## References

[ece34860-bib-0001] Adis, J. , Basset, Y. , Floren, A. , Hammond, P. M. , & Eduard, L. K. (1998). Canopy fogging of an overstory tree recommendations for standardization. Ecotropica, 4, 93–97.

[ece34860-bib-0002] Almeida‐Neto, M. , Guimarães, P. , Guimarães Jr, P. R. , Loyola, R. D. , & Ulrich, W. (2008). A consistent metric for nestedness analysis in ecological systems : Reconciling concept and measurement. Oikos, 117, 1227–1239. 10.1111/j.2008.0030-1299.16644.x

[ece34860-bib-0003] Almeida‐Neto, M. , & Ulrich, W. (2011). A straightforward computational approach for measuring nestedness using quantitative matrices. Environmental Modelling and Software, 26, 173–178. 10.1016/j.envsoft.2010.08.003

[ece34860-bib-0004] Bascompte, J. , Jordano, P. , Melián, C. J. , & Olesen, J. M. (2003). The nested assembly of plant‐animal mutualistic networks. Proceedings of the National Academy of Sciences of the United States of America, 100, 9383–9387. 10.1073/pnas.1633576100 12881488 PMC170927

[ece34860-bib-0005] Beckett, S. J. (2016). Improved community detection in weighted bipartite networks. Royal Society Open Science, 3, 140536 10.1098/rsos.140536 26909160 PMC4736915

[ece34860-bib-0006] Berger, A. (1992). Larval movements of Chilo partellus (Lepidoptera: Pyralidae ) within and between plants : Timing, density responses and survival. Bulletin of Entomological Research, 82, 441–448. 10.1017/S0007485300042498

[ece34860-bib-0007] Blüthgen, N. , Menzel, F. , & Blüthgen, N. (2006). Measuring specialization in species interaction networks. BMC Ecology, 6, 9 10.1186/1472-6785-6-9 16907983 PMC1570337

[ece34860-bib-0008] Braley, M. , Goldsworthy, S. D. , Page, B. , Steer, M. , & Austin, J. J. (2010). Assessing morphological and DNA‐based diet analysis techniques in a generalist predator, the arrow squid Nototodarus gouldi. Molecular Ecology Resources, 10, 466–474. 10.1111/j.1755-0998.2009.02767.x 21565046

[ece34860-bib-0009] Brousseau, P. M. , Gravel, D. , & Handa, I. T. (2018). Trait matching and phylogeny as predictors of predator–prey interactions involving ground beetles. Functional Ecology, 32, 192–202. 10.1111/1365-2435.12943

[ece34860-bib-0010] Burns, A. E. , Taylor, G. S. , Watson, D. M. , & Cunningham, S. A. (2015). Diversity and host specificity of Psylloidea (Hemiptera) inhabiting box mistletoe, Amyema miquelii (Loranthaceae) and three of its host Eucalyptus species. Austral Entomology, 54, 306–314. 10.1111/aen.12123

[ece34860-bib-0011] Delmas, E. , Besson, M. , Brice, M.‐H. , Burkle, L. , Riva, G. V. D. , Fortin, M.‐J. , … Poisot, T. (2019). Analyzing ecological networks of species interactions. Biological Reviews of the Cambridge Philosophical Society, 94, 16–36. 10.1101/112540 29923657

[ece34860-bib-0012] Derocles, S. A. P. , Evans, D. M. , Nichols, P. C. , Evans, S. A. , & Lunt, D. H. (2015). Determining plant‐leaf miner‐parasitoid interactions: A DNA barcoding approach. PLoS ONE, 10, e0117872 10.1371/journal.pone.0117872 25710377 PMC4339730

[ece34860-bib-0013] Derocles, S. A. P. , Le Ralec, A. , Besson, M. M. , Maret, M. , Walton, A. , Evans, D. M. , & Plantegenest, M. (2014). Molecular analysis reveals high compartmentalization in aphid‐primary parasitoid networks and low parasitoid sharing between crop and noncrop habitats. Molecular Ecology, 23, 3900–3911. 10.1111/mec.12701 24612360

[ece34860-bib-0014] Dormann, C. F. , Fründ, J. , Blüthgen, N. , & Gruber, B. (2009). Indices, graphs and null models : Analyzing bipartite ecological networks. The Open Ecology Journal, 2, 7–24. 10.2174/1874213000902010007

[ece34860-bib-0015] Dyer, L. A. , Singer, M. S. , Lill, J. T. , Stireman, J. O. , Gentry, G. L. , Marquis, R. J. , … Coley, P. D. (2007). Host specificity of Lepidoptera in tropical and temperate forests. Nature, 448, 696–699. 10.1038/nature05884 17687325

[ece34860-bib-0016] Erickson, D. L. , Reed, E. , Ramachandran, P. , Bourg, N. A. , McShea, W. J. , & Ottesen, A. (2017). Reconstructing a herbivore’s diet using a novel rbcL DNA mini‐barcode for plants. AoB Plants, 9, plx015 10.1093/aobpla/plx015 28533898 PMC5434754

[ece34860-bib-0017] Erwin, T. L. (1982). Tropical forests: Their richness in Coleoptera and other arthropod species. The Coleopterists Bulletin, 36, 74–75.

[ece34860-bib-0018] Evans, D. M. , Kitson, J. J. N. , Lunt, D. H. , Straw, N. A. , & Pocock, M. J. O. (2016). Merging DNA metabarcoding and ecological network analysis to understand and build resilient terrestrial ecosystems. Functional Ecology, 30, 1904–1916. 10.1111/1365-2435.12659

[ece34860-bib-0019] Fitzgerald, T. D. , Casey, T. , & Joos, B. (1988). Daily foraging schedule of field colonies of the eastern tent caterpillar Malacosoma americanum. Oecologia, 76, 574–578. 10.1007/BF00397873 28312411

[ece34860-bib-0020] Forister, M. L. , Novotny, V. , Panorska, A. K. , Baje, L. , Basset, Y. , Butterill, P. T. , … Dyer, L. A. (2015). The global distribution of diet breadth in insect herbivores. Proceedings of the National Academy of Sciences, 112, 442–447. 10.1073/pnas.1423042112 PMC4299246 25548168

[ece34860-bib-0021] Frederick, K. H. , & Gering, J. C. (2006). A field study of host tree associations of an exotic species, the asiatic oak weevil [Cyrtepistomus castaneus (Roelofs 1873), Coleoptera : Curculionidae]. American Midland Naturalist, 155, 11–18. 10.1674/0003-0031(2006)155[0011:AFSOHT]2.0.CO;2

[ece34860-bib-0022] García‐Robledo, C. , Erickson, D. L. , Staines, C. L. , Erwin, T. L. , & Kress, W. J. (2013). Tropical plant‐herbivore networks: Reconstructing species interactions using DNA barcodes. PLoS ONE, 8, e52967 10.1371/journal.pone.0052967 23308128 PMC3540088

[ece34860-bib-0023] Hajibabaei, M. , Janzen, D. H. , Burns, J. M. , Hallwachs, W. , & Hebert, P. D. N. (2006). DNA barcodes distinguish species of tropical Lepidoptera. Proceedings of the National Academy of Sciences of the United States of America, 103, 968–971. 10.1073/pnas.0510466103 16418261 PMC1327734

[ece34860-bib-0024] Hebert, P. D. N. , Cywinska, A. , Ball, S. L. , & DeWaard, J. R. (2003). Biological identifications through DNA barcodes. Proceedings of the Royal Society B: Biological Sciences, 270(1512), 313–321. 10.1098/rspb.2002.2218 PMC1691236 12614582

[ece34860-bib-0025] Hebert, P. D. N. , DeWaard, J. R. , & Landry, J.‐F. (2010). DNA barcodes for 1/1000 of the animal kingdom. Biology Letters, 6, 359–362. 10.1098/rsbl.2009.0848 20015856 PMC2880045

[ece34860-bib-0026] Jacquet, C. , Moritz, C. , Morissette, L. , Legagneux, P. , Massol, F. , Archambault, P. , & Gravel, D. (2016). No complexity – stability relationship in empirical ecosystems. Nature Communications, 7, 12573 10.1038/ncomms12573 PMC4999500 27553393

[ece34860-bib-0027] Jurado‐Rivera, J. A. , Vogler, A. P. , Reid, C. A. , Petitpierre, E. , & Gómez‐Zurita, J. (2009). DNA barcoding insect‐host plant associations. Proceedings of the Royal Society B: Biological Sciences, 276, 639–648. 10.1098/rspb.2008.1264 PMC2660938 19004756

[ece34860-bib-0028] Kaartinen, R. , Stone, G. N. , Hearn, J. , Lohse, K. , & Roslin, T. (2010). Revealing secret liaisons: DNA barcoding changes our understanding of food webs. Ecological Entomology, 35, 623–638. 10.1111/j.1365-2311.2010.01224.x

[ece34860-bib-0029] Kajtoch, Ł. (2014). A DNA metabarcoding study of a polyphagous beetle dietary diversity: The utility of barcodes and sequencing techniques. Folia Biologica, 62, 223–234. 10.3409/fb62 25412510

[ece34860-bib-0030] Kartzinel, T. R. , Chen, P. A. , Coverdale, T. C. , Erickson, D. L. , Kress, W. J. , Kuzmina, M. L. , … Pringle, R. M. (2015). DNA metabarcoding illuminates dietary niche partitioning by African large herbivores. Proceedings of the National Academy of Sciences, 112, 8019–8024. 10.1073/pnas.1503283112 PMC4491742 26034267

[ece34860-bib-0031] Montoya, J. M. , Pimm, S. L. , & Solé, R. V. (2006). Ecological networks and their fragility. Nature, 442, 259–264. 10.1038/nature04927 16855581

[ece34860-bib-0032] Morgulis, A. , Coulouris, G. , Raytselis, Y. , Madden, T. L. , Agarwala, R. , & Schäffer, A. A. (2008). Database indexing for production MegaBLAST searches. Bioinformatics, 24, 1757–1764. 10.1093/bioinformatics/btn322 18567917 PMC2696921

[ece34860-bib-0033] Navarro, S. P. , Jurado‐Rivera, J. A. , Gómez-Zurita, J. , Lyal, C. H. C. , & Vogler, A. P. (2010). DNA profiling of host‐herbivore interactions in tropical forests. Ecological Entomology, 35, 18–32. 10.1111/j.1365-2311.2009.01145.x

[ece34860-bib-0034] Novotny, V. , Basset, Y. , Miller, S. E. , Drozd, P. , & Cizek, L. (2002). Host specialization of leaf‐chewing insects in a New Guinea rainforest. Journal of Animal Ecology, 71, 400–412. 10.1046/j.1365-2656.2002.00608.x

[ece34860-bib-0035] Novotny, V. , Basset, Y. , Miller, S. E. , Weiblen, G. D. , Bremer, B. , Cizek, L. , & Drozd, P. (2002). Low host specificity of herbivorous insects in a tropical forest. Nature, 416, 841–844. 10.1038/416841a 11976681

[ece34860-bib-0036] Nuwagaba, S. , Zhang, F. , & Hui, C. (2015). A hybrid behavioural rule of adaptation and drift explains the emergent architecture of antagonistic networks. Proceedings of the Royal Society B: Biological Sciences, 282, 20150320 10.1098/rspb.2015.0320 PMC4424652 25925104

[ece34860-bib-0037] Olesen, J. M. , Bascompte, J. , Dupont, Y. L. , & Jordano, P. (2007). The modularity of pollination networks. Proceedings of the National Academy of Sciences, 104, 19891–19896. 10.1073/pnas.0706375104 PMC2148393 18056808

[ece34860-bib-0038] Post, R. J. , Flook, P. K. , & Millest, A. L. (1993). Methods for the preservation of insects for DNA studies. Biochemical Systematics and Ecology, 21, 85–92. 10.1016/0305-1978(93)90012-G

[ece34860-bib-0039] Pumariño, L. , Alomar, O. , & Agustí, N. (2011). Development of specific ITS markers for plant DNA identification within herbivorous insects. Bulletin of Entomological Research, 101, 271–276. 10.1017/S0007485310000465 21092379

[ece34860-bib-0040] Šigut, M. , Kostovčík, M. , Šigutova, H. , Hulcr, J. , Drozd, P. , & Hrček, J. (2017). Performance of DNA metabarcoding, standard barcoding, and morphological approach in the identification of host – parasitoid interactions. Plose One, 12, e0187803 10.1371/journal.pone.0187803 PMC5728528 29236697

[ece34860-bib-0041] Smith, M. A. , Eveleigh, E. S. , Mccann, K. S. , Merilo, M. T. , Mccarthy, P. C. , & Van Rooyen, k. (2011). Barcoding a quantified food web : Crypsis, concepts, ecology and hypotheses. PLoS ONE, 6, e14424 10.1371/journal.pone.0014424 21754977 PMC3130735

[ece34860-bib-0042] Stork, N. E. (1987). Guild structure of arthropods from Bornean rain forest trees. Ecological Entomology, 12, 69–80. 10.1111/j.1365-2311.1987.tb00986.x

[ece34860-bib-0043] Strutzenberger, P. , Brehm, G. , & Fiedler, K. (2011). DNA barcoding‐based species delimitation increases species count of Eois (Geometridae) moths in a well‐studied tropical mountain forest by up to 50%. Insect Science, 18, 349–362. 10.1111/j.1744-7917.2010.01366.x

[ece34860-bib-0044] Thébault, E. , & Fontaine, C. (2010). Stability of ecological communities and the architecture of mutualistic and trophic networks. Science, 329, 853–856. 10.1126/science.1188321 20705861

[ece34860-bib-0045] Wilson, E. E. , Sidhu, C. S. , Levan, K. E. , & Holway, D. A. (2010). Pollen foraging behaviour of solitary Hawaiian bees revealed through molecular pollen analysis. Molecular Ecology, 19, 4823–4829. 10.1111/j.1365-294X.2010.04849.x 20958818

[ece34860-bib-0046] Wirta, H. K. , Hebert, P. D. N. , Kaartinen, R. , Prosser, S. W. , Várkonyi, G. , & Roslin, T. (2014). Complementary molecular information changes our perception of food web structure. Proceedings of the National Academy of Sciences, 111, 1885–1890. 10.1073/pnas.1316990111 PMC3918760 24449902

[ece34860-bib-0047] Zhang, Z. , Schwartz, S. , Wagner, L. , & Miller, W. (2000). A greedy algorithm for aligning DNA sequences. Journal of Computational Biology, 7, 203–214. 10.1089/10665270050081478 10890397

