# Supplementary material for: Seeing is believing? Comparing plant–herbivore networks constructed by field co‐occurrence and DNA barcoding methods for gaining insights into network structures
Source: Ecol Evol. 2019 Feb 7;9(4):1764–76. doi: 10.1002/ece3.4860 (PMC6392357; doi:10.1002/ece3.4860)
Supplement: Supplementary file 1 [file ECE3-9-1764-s001.docx]

**Seeing is believing? Comparing plant-herbivore networks constructed by field observation and DNA barcoding methods for gaining insights into network structures**

Running head: DNA-based network and observation-based network

Chunchao Zhu^1,2^, Dominique Gravel^3^ and Fangliang He^2,4^

^1^State Key Laboratory of Biocontrol and School of Life Sciences, Sun Yat-sen University, Guangzhou, China

^2^ECNU-Alberta Joint Lab for Biodiversity Study, Tiantong National Station for Forest Research, School of Ecology and Environmental Sciences, East China Normal University, Shanghai, China

^3^Département de Biologie, Université de Sherbrooke, Sherbrooke, Quebec, Canada

^4^Department of Renewable Resources, University of Alberta, Edmonton, Alberta, Canada

Correspondence author email: [fhe@ualberta.ca](mailto:fhe@ualberta.ca)

SUPPORTING INFORMATION

FIGURE S1 Abundance distribution of Lepidoptera larvae fed on nearest food resource over the distance between the nearest food resource and the fogged (sampled) tree. Food resource of Lepidoptera larvae are identified by the molecular method. This plot displays the matched nearest food plant identified by molecular method in a radius of 50 meter from the sampled tree. Due to lacking mapped data, we didn’t locate food resources identified as additional plant taxa (bamboos, bryophytes and lianas) and a portion of low species resolution taxa by molecular approach.

FIGURE S2 Modularity (A) and nestedness (B) of the observation neworks and molecular networks. One full observation network (based on 2235 caterpillar individuals) and five subwebs documented by removing rare links ranging from 1 to 5 from the full observation network were constructed. The modulariy and nestedness of the six observation networks including minimum interaction number ranging from 1 to 6 were compared with molecular network. The molecular network was constructed based on 795 caterpillars whose diets were recovered by the DNA barcoding method. The dashed line denotes the molecular network and the black point denotes observation network.

TABLE S1 The detail of barcode primers used in this study.

| DNA barcode regions | | Primer name | Sequences ( 5’-3’) | Annealing  temp (℃) | References |
| --- | --- | --- | --- | --- | --- |
| rbcLa | rbcLa-f | | ATGTCACCACAAACAGAGACTAAAGC | 50 | (Kress et al., 2009) |
|  | rbcLa-rev | | GTAAAATCAAGTCCACCRCG | 50 | (Kress et al., 2009) |
| trnL | trnLc | | CGAAATCGGTAGACGCTACG | 54 | (Taberlet, Gielly, Pautou, & Bouvet, 1991) |
|  | trnLd | | GGGGATAGAGGGACTTGAAC | 54 | (Taberlet et al., 1991) |
| ITS2 | ITS2-S2F | | ATGCGATACTTGGTGTGAAT | 53 | (Chiou, Yen, Fang, Chen, & Lin, 2007) |
|  | ITS2-S3R | | GACGCTTCTCCAGACTACAAT | 53 | (Chiou et al., 2007) |
| HCO | | HCO2198 | TAAACTTCAGGGTGACCAAAAAATCA | 54 | (Folmer, Black, Hoeh, Lutz, & Vrijenhoek, 1994) |
| LCO | | LCO1490 | GGTCAACAAATCATAAAGATATTGG | 54 | (Folmer et al., 1994) |

TABLE S2 Host plant genera uniquely identified by the molecular method and the traditional observation method, respectively. Shown are also those genera commonly identified by both methods.

| Unique genera in molecular network | Unique genera in observation network | Common genera in two networks |
| --- | --- | --- |
| *Pseudosasa* | *Meliosma* | *Altingia* |
| *Calamus* | *Pittosporum* | *Antidesma* |
| *Pinus* | *Saurauia* | *Ardisia* |
| *Millettia* |  | *Artocarpus* |
| *Acacia* |  | *Callicarpa* |
| *Ixonanthes* |  | *Camellia* |
| *Embelia* |  | *Castanopsis* |
| *Helicia* |  | *Cinnamomum* |
| *Tetrastigma* |  | *Corylopsis* |
| *Mussaenda* |  | *Craibiodendron* |
| *Strychnos* |  | *Cryptocarya* |
| *Cololejeunea* |  | *Cyclobalanopsis* |
| *Rourea* |  | *Diplospora* |
| *Pleioblastus* |  | *Distylium* |
| *Gnetum* |  | *Elaeocarpus* |
| *Elaeagnus* |  | *Engelhardtia* |
| *Acer* |  | *Enkianthus* |
| *Desmos* |  | *Eriobotrya* |
| *Scurrula* |  | *Eurya* |
| *Ilex* |  | *Ficus* |
| *Illicium* |  | *Gardenia* |
| *Wendlandia* |  | *Stewartia* |
| *Fissistigma* |  | *Homalium* |
| *Pluvianthus* |  | *Itea* |
| *Erycibe* |  | *Laurocerasus* |
| *Strophanthus* |  | *Lindera* |
| *Myrica* |  | *Lithocarpus* |
| *Adina* |  | *Litsea* |
| *Garcinia* |  | *Machilus* |
| *Dalbergia* |  | *Microtropis* |
| *Diospyros* |  | *Neolitsea* |
| *Vitex* |  | *Ormosia* |
| *Helixanthera* |  | *Photinia* |
| *Michelia* |  | *Pithecellobium* |
| *Heteropanax* |  | *Randia* |
|  |  | *Myrsine* |
|  |  | *Rhododendron* |
|  |  | *Schefflera* |
|  |  | *Schima* |
|  |  | *Sinosideroxylon* |
|  |  | *Sloanea* |
|  |  | *Symplocos* |
|  |  | *Syzygium* |
|  |  | *Tarenna* |
|  |  | *Ternstroemia* |
|  |  | *Tutcheria* |
|  |  | *Xanthophyllum* |

TABLE S3 Host plant species uniquely identified by the molecular method and the traditional observation method, respectively. Shown are also those species commonly identified by both methods.

| Unique species in molecular network | Shared species in two networks | Unique species in observation networks |
| --- | --- | --- |
| *Pleioblastus maculatus* | *Altingia chinensis* | *Camellia cordifolia* |
| *Calamus sp* | *Antidesma venosum* | *Castanopsis carlesii* |
| *Pinus massoniana* | *Ardisia quinquegona* | *Cyclobalanopsis bambusaefolia* |
| *Millettia dielsiana* | *Artocarpus styracifolius* | *Cyclobalanopsis chungii* |
| *Acacia sinuata* | *Callicarpa peichieniana* | *Cyclobalanopsis fleuryi* |
| *Ixonanthes chinensis* | *Camellia caudata* | *Cyclobalanopsis hui* |
| *Embelia sp* | *Camellia semiserrata* | *Diplospora dubia* |
| *Symplocos adenophylla* | *Camellia sinensis* | *Eurya hebeclados* |
| *Helicia kwangtungensis* | *Castanopsis fissa* | *Litsea rotundifolia var. oblongifolia* |
| *Tetrastigma obovatum* | *Castanopsis nigrescens* | *Machilus velutina* |
| *Mussaenda pubescens* | *Cinnamomum burmanni* | *Meliosma rigida var. pannosa* |
| *Symplocos wikstroemiifolia* | *Cinnamomum porrectum* | *Pittosporum glabratum* |
| *Strychnos cathayensis* | *Corylopsis sinensis* | *Saurauia tristyla* |
| *Cololejeunea ocelloides* | *Craibiodendron stellatum* | *Symplocos anomala* |
| *Rourea minor* | *Cryptocarya concinna* | *Symplocos congesta* |
| *Gnetum parvifolium* | *Distylium racemosum* | *Symplocos laurina* |
| *Cinnamomum appelianum* | *Elaeocarpus chinensis* |  |
| *Elaeagnus sp* | *Elaeocarpus decipiens* |  |
| *Acer tutcheri* | *Engelhardtia fenzlii* |  |
| *Syzygium kwangtungense* | *Enkianthus serrulatus* |  |
| *Pseudosasa sp* | *Eriobotrya fragrans* |  |
| *Desmos chinensis* | *Ficus variolosa* |  |
| *Cyclobalanopsis spch* | *Gardenia jasminoides* |  |
| *Cyclobalanopsis spbf* | *Stewartia villosa* |  |
| *Scurrula sp* | *Homalium cochinchinense* |  |
| *Diplospora fruticosa* | *Itea chinensis* |  |
| *Ilex memecylifolia* | *Laurocerasus phaeosticta* |  |
| *Illicium dunnianum* | *Lindera chunii* |  |
| *Wendlandia uvariifolia* | *Lithocarpus calophyllus* |  |
| *Eurya sp* | *Lithocarpus litseifolius* |  |
| *Fissistigma oldhamii* | *Lithocarpus lohangwu* |  |
| *Ardisia punctata* | *Lithocarpus uvariifolius* |  |
| *Pluvianthus squarrosus* | *Litsea acutivena* |  |
| *Erycibe obtusifolia* | *Litsea elongata* |  |
| *Strophanthus divaricatus* | *Litsea greenmaniana* |  |
| *Myrica rubra* | *Machilus breviflora* |  |
| *Adina pilulifera* | *Microtropis gracilipes* |  |
| *Garcinia multiflora* | *Neolitsea phanerophlebia* |  |
| *Dalbergia hancei* | *Ormosia fordiana* |  |
| *Diospyros sp* | *Ormosia glaberrima* |  |
| *Machilus litseifolia* | *Ormosia pachycarpa* |  |
| *Vitex quinata* | *Photinia prunifolia* |  |
| *Helixanthera parasitica* | *Pithecellobium lucidum* |  |
| *Ilex fragilis* | *Randia canthioides* |  |
| *Michelia skinneriana* | *Myrsine seguinii* |  |
| *Heteropanax brevipedicellatus* | *Rhododendron simsii* |  |
|  | *Schefflera octophylla* |  |
|  | *Schima superba* |  |
|  | *Sinosideroxylon wightianum* |  |
|  | *Sloanea sinensis* |  |
|  | *Symplocos lancifolia* |  |
|  | *Syzygium hancei* |  |
|  | *Tarenna mollissima* |  |
|  | *Ternstroemia gymnanthera* |  |
|  | *Tutcheria championi* |  |
|  | *Xanthophyllum hainanense* |  |

TABLE S4 Comparision of node number between the molecular networks and observation networks constructed by each method. Seven molecular netwoks were constructed using the combination of one, two, three DNA markers, respectively. Observation networks were constructed with same larval sample as the molecular network constructed by each combination of DNA markers. Diet association in an observation network was determined by fogging records. Sample size represents the number of Lepidoptera larval individuals. Plant node in each of molecular networks constructed by the combination of different DNA markers was assigned as the lowest taxonomic level.

|  |  | *rbcL* | *trnL* | ITS | *rbcLtrnL* | *rbcl*ITS | *trnL*ITS | *rbcLtrnL*ITS |
| --- | --- | --- | --- | --- | --- | --- | --- | --- |
| Molecular networks | No. of plant node | 90 | 56 | 37 | 97 | 98 | 71 | 102 |
|  | No. of insect node | 190 | 162 | 83 | 217 | 199 | 189 | 239 |
|  | Sample size | 567 | 408 | 218 | 692 | 638 | 506 | 795 |
| Observation networks | No. of Plant node | 72 | 67 | 37 | 72 | 72 | 71 | 72 |
|  | No. of insect node | 190 | 162 | 83 | 217 | 199 | 180 | 239 |
|  | Sample size | 567 | 408 | 218 | 692 | 638 | 506 | 795 |

**REFERENCES**

Chiou, S. J., Yen, J. H., Fang, C. L., Chen, H. L., & Lin, T. Y. (2007). Authentication of medicinal herbs using PCR-amplified ITS2 with specific primers. *Planta Medica*, *73*, 1421–1426.

Folmer, O., Black, M., Hoeh, W., Lutz, R., & Vrijenhoek, R. (1994). DNA primers for amplification of mitochondrial cytochrome c oxidase subunit I from diverse metazoan invertebrates. *Molecular Marine Biology and Biotechnology*, *3*, 294–299. doi:10.1371/journal.pone.0013102

Kress, W. J., Erickson, D. L., Jones, F. A., Swenson, N. G., Perez, R., Sanjur, O., & Bermingham, E. (2009). Plant DNA barcodes and a community phylogeny of a tropical forest dynamics plot in Panama. *Proceedings of the National Academy of Sciences*, *106*, 18621–18626. doi:10.1073/pnas.0909820106

Taberlet, P., Gielly, L., Pautou, G., & Bouvet, J. (1991). Universal primers for amplification of three non-coding regions of chloroplast DNA. *Plant Molecular Biology*, *17*, 1105–1109. doi:10.1007/BF00037152
